# Salivary and Serum Interleukin-10, C-Reactive Protein, Mean Platelet Volume, and CRP/MPV Ratio in the Diagnosis of Late-Onset Neonatal Sepsis in Full-Term Neonates

**DOI:** 10.1155/2021/4884537

**Published:** 2021-10-12

**Authors:** Ahmed Omran, Hazem Sobh, Mohamed Osama Abdalla, Sonya El-Sharkawy, Ahmed R. Rezk, Abdelmoneim Khashana

**Affiliations:** ^1^Department of Pediatrics & Neonatology, Faculty of Medicine, Suez Canal University, Ismailia, Egypt; ^2^Clinical Pathology Department, Faculty of Medicine, Suez Canal University, Ismailia, Egypt; ^3^Departments of Pediatrics & Neonatology, Faculty of Medicine, Port Said University, Port Said, Egypt; ^4^Department of Pediatrics, Ain Shams University, Cairo, Egypt

## Abstract

Salivary markers could serve as potential noninvasive markers in the diagnosis of neonatal infections. We aimed to investigate the diagnostic role of salivary and serum interleukin 10 (IL-10), C-reactive protein (CRP), mean platelet volume (MPV), and CRP/MPV ratio in the diagnosis of late-onset neonatal sepsis in full-term neonates. Seventy full-term neonates were enrolled in this prospective case-control study, 35 with late-onset neonatal sepsis, and 35 controls. Salivary IL-10, serum IL-10, and CRP concentrations were measured by enzyme-linked immunosorbent assay (ELISA). Complete blood (CBC) count was measured by an automated blood cell counter. The salivary IL-10, serum IL-10, CRP, MPV, and CRP/MPV ratio levels were much higher in neonates with late-onset sepsis than in control (220 ± 150 vs. 18 ± 9 pg/ml, *P* < 0.001), (316 ± 198 vs. 23.7 ± 14 pg/ml, *P* < 0.001), (78.2 ± 34 vs. 3.3 ± 1.7 mg/L, *P* < 0.001), (11.2 ± 0.9 vs. 8.6 ± 0.4 fL), and (7.08 ± 3.3 vs. 0.4 ± 0.2, *P* < 0.001), respectively. At the cutoff point of >31 pg/ml, salivary IL-10 showed 97.1% sensitivity and 94.3% specificity. Serum IL-10 at a cutoff value of ≥33.6 pg/ml had a sensitivity of 97.1% and specificity of 80%. MPV showed a sensitivity of 100% and specificity of 94.4% at a cutoff value ≥ 9.2 fL. CRP/MPV ratio showed a sensitivity of 100% and specificity of 97.1% at a cutoff value > 0.9. Salivary and serum IL-10 showed a positive correlation with CRP and CRP/MPV ratio in septic neonates. The current study shows for the first time that both salivary IL-10 and CRP/MPV showed statistically significant differences between neonates with late-onset sepsis and controls. Accordingly, salivary IL-10 could serve as a potential noninvasive biomarker for the diagnosis of late-onset sepsis in full-term neonates.

## 1. Introduction

Despite marked improvement in neonatal care, sepsis remains an important contributor to the neonatal morbidity and mortality worldwide [1, 2]. Neonatal sepsis can be defined both clinically and/or microbiologically as a systemic inflammatory response to infection with the production and release of a wide range of inflammatory mediators. Early detection of neonatal sepsis is still a very challenging task because of its nonspecific clinical presentation and difficulty to differentiate from noninfectious conditions [3, 4]. The gold standard to diagnose neonatal sepsis is blood culture which may be time-consuming and has limited sensitivity.

Interleukin-10 (IL-10) is a key anti-inflammatory cytokine produced by many different kinds of immune cells and has a crucial role in preventing inflammatory and autoimmune pathologies [5–7]. The expression of anti-inflammatory cytokines including IL-10 is predominately occurring during the second phase of neonatal sepsis which reflects the upregulation of immunosuppressive mechanisms [8]. Therefore, IL-10 plays an important role in the early diagnosis of late-onset neonatal sepsis [9–12].

Saliva is an excellent noninvasive and easily accessible biofluid that represents an alternative to serum testing to assess and monitor critically ill neonates. Salivary biomarkers were reported to detect infections in full-term and preterm infants [13–18].

C-reactive protein (CRP) is an established marker of systemic inflammation and one of the most commonly used and extensively studied markers for the diagnosis of neonatal sepsis [3, 15, 17, 19]. High mean platelet volume (MPV) is a sign of platelet activation. In clinical practice, MPV could be used as an indicator for the early diagnosis of neonatal sepsis [15, 20–23]. Several studies have examined the potential correlation between CRP and MPV values in patients with inflammatory states [24–27]. But only one study investigated the CRP/MPV ratio in children with pneumonia [28]. Various other ratios are being used in the diagnosis of neonatal sepsis, which includes CRP/albumin ratio [29], neutrophil–lymphocyte ratio [15, 30–33], and platelet to lymphocyte ratio [34].

To the best of our knowledge, this is the first study to evaluate the applicability of salivary IL-10 and CRP/MPV ratio as diagnostic markers in full-term neonates with late-onset sepsis.

## 2. Materials and Methods

### 2.1. Study Population

A prospective case-control study was conducted at the neonatal intensive care unit (NICU) of the Suez Canal University Hospital, Ismailia, Egypt, between January 2018 and January 2019. Thirty-five neonates with late-onset sepsis and 35 control neonates were enrolled. The protocol was approved by the Institutional Review and Research Ethics Boards of the Faculty of Medicine, Suez Canal University. Written informed consent was obtained from the parents of all neonates in the study.

Full-term neonates admitted in the NICU, who were suspected to have late-onset sepsis (onset after 72 h of birth), were enrolled in the study. The diagnosis of clinical sepsis was reached by history, clinical findings, laboratory findings, and blood culture. A clinical sepsis score was created based on a score defined by Töllner [35]. This included the presence of three or more of the following categories of clinical signs: (1) temperature instability (hyperthermia and hypothermia); (2) cardiovascular alterations (tachycardia, bradycardia, poor perfusion, and hypotension); (3) respiratory alterations (tachypnea, grunting, intercostal retractions, cyanosis, and apnea); (4) gastrointestinal alterations (abdominal distension and feeding intolerance); and (5) neurologic alterations (lethargy, hypotonia, and seizures).

Neonates with early-onset sepsis, preterm, and neonates with confirmed pneumonia or other inflammatory conditions, metabolic disorders, intrauterine growth restriction, CNS malformations, chromosomal abnormalities, or birth asphyxia were excluded from the study.

The controls were age- and sex-matched neonates with no clinical or laboratory findings of sepsis and were followed up in our hospital for non-infectious, unconjugated hyperbilirubinemia, and males evaluated before circumcision.

Sample size: the sample size was calculated using the following formula [36].

(1) $$\begin{array}{c} n={\left[\frac{Z_{\alpha /2}}{E}\right]}^{2}*P\left(1-P\right), \end{array}$$

where *n* is the sample size. *Z*_*α*/2_ = 1.96 is the critical value that divides the central 95% of the *Z* distribution from the 5% in the tail. *E* is the margin of error/width of confidence interval = 5%. *P* is the prevalence/proportion in the study group = 2.23% [37].

The final sample size was 70 (35 cases and 35 controls).

### 2.2. Saliva Collection

Salivary samples were collected at 7 am because of the independent diurnal rhythms of salivary cytokines and 1 hour before feeding to avoid milk contamination [38]. Collection of saliva was performed as mentioned in previously published researches [14, 15, 39]. After collection, samples were put in polypropylene vials to avoid contamination and analytic retention faults. Then samples were sent to the laboratory immediately and stored at -20°C until batch analysis.

### 2.3. Laboratory Workup

For each patient, 2 ml of venous blood was collected in ethylenediaminetetraacetic acid (EDTA) and plain tubes. EDTA samples were used for complete blood count (CBC) using the Sysmex XN-550 automatic cell counter (Sysmex Corp., Kobe, Japan). Samples were analyzed within 1 h after sampling to avoid platelet swelling. Blood samples collected in plain tubes were left for complete coagulation at room temperature, and then serum was separated by centrifugation at 3000 rpm for 10 minutes. Serum CRP concentration was assessed using Cobas 6000 analyzer (Roche, Mannheim, Germany). Serum and salivary IL-10 were measured by enzyme-linked immunosorbent assay (ELISA) kit (E0056h, EIAab, Shanghai, China) according to the manufactures' instructions. All samples were collected before starting antibiotic therapy.

Blood cultures were performed for each infant with suspected sepsis at the time of admission and before antibiotic therapy. 1 ml of blood was placed in a pediatric blood culture bottle. Cultures were done using the BacT/Alert system (BioMérieux, Durham, NC, US). Bacterial identification of positive samples was done by standardized biochemical tests, and antibiotic susceptibility was assessed according to the Clinical and Laboratory Standards Institute method.

### 2.4. Statistical Analysis

The statistical analysis was performed using SPSS for Windows statistical package, version 20 (SPSS, Chicago, IL, USA). The differences between groups regarding nonparametric quantitative data were assessed by Mann–Whitney *U*-test. The Chi-square test was used for testing significant differences of qualitative variables. The sensitivity, specificity, optimal serum, and salivary IL-10 cut points were determined using the receiver operating characteristic (ROC) curve. For all statistical analyses, the level of considered significance was <0.05.

## 3. Results

### 3.1. Population Characteristics, Prenatal, and Clinical Presentation

**Figure 1:**
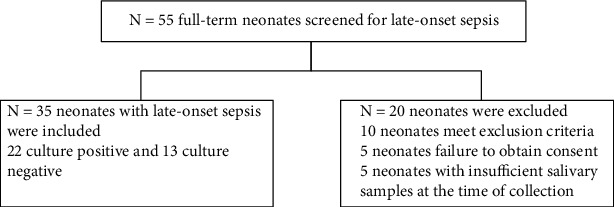
Patient's flow chart.

During the study period, 90 full-term neonates were included in study 55 with suspected sepsis from which only 35 matched the inclusion criteria and 35 controls (Figure 1). The mean age for sepsis and controls were (13 ± 6 and 11 ± 2) days, respectively, males constitute 60% of the sepsis group and 54.3% of the controls with no statistically significant difference. Regarding weight, gestational age, and mode of delivery, there were also no statistically significant differences between both groups (*P* value > 0.05).

Regarding the prenatal data, there were no statistically significant differences between both groups in the history of premature rupture of membrane (PROM), maternal fever, and maternal urinary tract infection (UTI) (*P* value > 0.05).

Lethargy and temperature instability were the most presenting clinical findings in the sepsis group (85.7% and 77.1%, respectively), followed by poor perfusion and hypotension presented in 54.3%, and DIC presented in 25.7%.

### 3.2. Pathogen Distribution

Blood cultures were positive in 22 infants (62.8%) in the sepsis group. The identified bacteria included Klebsiella pneumonia (*n* = 8), Escherichia coli (*n* = 5), Staphylococcus aureus (*n* = 5), and mixed infection (*n* = 4).

### 3.3. Comparison of Salivary and Serum IL-10, CRP/MPV Ratio, and Other Markers between the Two Groups

As presented in Table 1, laboratory results between the sepsis group and the control group were compared. Septic neonates had significantly lower platelet count compared to the control group (*P* = 0.012). Septic neonates had significantly higher MPV (mean 11.2 ± 0.9 vs. 8.6 ± 0.4 fL, *P* < 0.001), CRP (mean 78.2 ± 34 vs. 3.3 ± 1.7 mg/L, *P* < 0.001), and CRP/MPV ratio (7.08 ± 3.3 vs. 0.4 ± 0.2, *P* < 0.001). Both serum and salivary IL-10 were also significantly higher in septic neonates (mean 316 ± 198 vs. 23.7 ± 14 pg/ml, *P* < 0.001) and (mean 220 ± 150 vs. 18 ± 9 pg/ml, *P* < 0.001), respectively. There was no significant difference in total leucocyte count.

### 3.4. Comparison of Salivary and Serum IL-10, CRP/MPV Ratio, and Other Markers in Blood Culture Positive and Negative Septic Cases

In septic neonates, there were no significant differences in the results of MPV, CRP, CRP/MPV ratio, serum, and salivary IL-10 between blood culture positive and negative septic cases (Table 2).

### 3.5. ROC Curve Analysis for Salivary and Serum IL-10, MPV, and CRP/MPV to Predict Late-Onset Sepsis

MPV showed a sensitivity of 100% and specificity of 94.3% at a cutoff value ≥ 9.2 fL. CRP/MPV ratio showed the highest sensitivity of 100% and specificity of 97.1% at a cutoff value ≥ 0.90. Serum IL-10 at a cutoff value ≥ 33.6 showed sensitivity of 97.1%, specificity 80%, PPV 82.9%, and NPV 96.6%. Salivary IL-10 at a cutoff value ≥ 31 showed very high sensitivity of 97.1%, specificity 94.3%, PPV 94.4%, and NPV 97.1% (Table 3).

### 3.6. Salivary and Serum IL-10 to Discriminate Serum CRP Values > 10 mg/L

**Figure 2:**
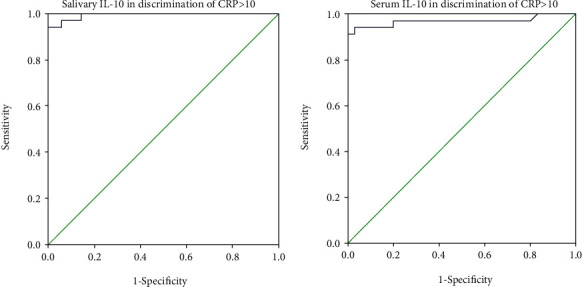
Salivary and serum IL-10 in discrimination of serum CRP values > 10 mg/L.

We also tested the ROC curves for serum and salivary IL-10 and their abilities to discriminate serum CRP values > 10 mg/L. ROC curves showed that both serum and salivary IL-10 have excellent ability to detect serum CRP values > 10 mg/L in septic neonates (Figure 2).

### 3.7. Correlation between Salivary and Serum IL-10 and Other Laboratory Markers

We also tested the correlation between salivary and serum IL-10 and other laboratory markers. There was a significant positive correlation between salivary IL-10 with both CRP (*r* = 0.356, *P* < 0.05) and CRP/MPV ratio (*r* = 0.390, *P* < 0.05). Also, there was a significantly positive correlation between serum IL-10 with TLC (*r* = 0.374, *P* < 0.05), CRP (*r* = 0.39, *P* < 0.05), and CRP/MPV ratio (*r* = 0.424, *P* < 0.05) Table 4.

## 4. Discussion

Neonatal sepsis is a major public health problem and the third leading cause of neonatal mortality. Although the great advances in neonatal medical care, many challenges remain in the diagnosis of neonatal sepsis. In this study, we evaluated the diagnostic value of salivary IL-10 as a noninvasive marker as well as serum IL-10, CRP, MPV, and CRP/MPV ratio in early diagnosis of late-onset neonatal sepsis in full-term neonates.

In high-risk infants, mortality rates in late-onset sepsis reach up to 18% [40]. Sick hospitalized neonates require laboratory tests and repeated blood sampling which exposed these fragile populations to the risk of pain, infection, cardiorespiratory instability, and anemia. There is an urgent need for a reliable and accurate noninvasive diagnostic biomarker for the early diagnosis of late-onset neonatal sepsis.

Salivary testing is a noninvasive alternate to blood for diagnosis and monitoring common neonatal morbidities, such as infections, and could vastly improve the care for neonates and positively affect their long-term clinical outcomes.

To the best of our knowledge, our study investigates, for the first time, salivary IL-10 as a noninvasive marker for the diagnosis of late-onset sepsis in full-term neonates. In our study, a salivary IL-10 value was significantly different between septic and control neonates and showed very high sensitivity and specificity. Sesso et al. [41] reported that salivary cytokines including salivary IL-10 could be detected in full-term neonates within the first hours after birth, and their levels decreased after 3 months. Recently, Chen et al. [18] investigated the relationship between salivary cytokines and neonatal bacterial infection in premature neonates; they found that the combination of blood sugar and salivary IL-6 could detect bacterial infection in preterm neonates.

In our study, the mean serum IL-10 was also significantly different between the septic and control neonates. Wang et al. [12], in just-published meta-analysis, found that IL-10 is a useful biomarker in the early diagnosis of neonatal sepsis. Its sensitivity, specificity, and diagnostic ability are excellent.

Other studies showed that the optimal cutoff value of IL-10 in the diagnosis of neonatal sepsis ranged from 3.8 to 49 pg/ml. The difference may be related to the different detection methods of IL-10 expression [9, 10, 42–44]. Zeitoun et al. [9] found the average expression of IL-10 in the late-onset neonatal sepsis group was 198.3 pg/ml. Furthermore, the expression level of IL-10 was positively correlated with the severity of neonatal sepsis. The more severe cases of neonatal sepsis had higher expression levels of IL-10, especially in low birth weight infants with sepsis [44–46].

In our study, there was no significant difference in the mean of salivary and serum IL-10 in culture-positive and culture-negative neonates. Zeitoun et al. [9] reported the same observation on serum IL-10 in culture-positive cases with a mean of 189.3 pg/ml compared with a mean of 198.3 pg/ml in culture-negative cases.

In our study, salivary and serum IL-10 showed a positive correlation with CRP and CRP/MPV ratio in septic neonates. Unexpectedly, Khaertynov et al. [8] observed no correlation between serum IL-10 and CRP in their study. This could return to different characteristics in the study population in the two studies.

Despite the detection of new markers, CRP is still one of the most expressively used acute phase reactants in the diagnosis of neonatal sepsis. Our study revealed a significant difference between septic and control neonates. In agreement with our results, other researchers observed the same findings [3, 15–17]. Also, salivary CRP was investigated as a noninvasive marker for the diagnosis of neonatal sepsis [15–17].

Our study showed that MVP was significantly different between septic and the control neonates. In support to our results, just published 2 meta-analyses reported significantly higher MPV in newborns with sepsis compared to healthy controls [22, 23]. Very close to our results, Milas et al. [22], in their meta-analysis, reported an optimal cutoff point of 9.28 fL for differentiating septic neonates from healthy controls.

Our study also investigates the value of CRP/MPV ratio in diagnosis of late-onset neonatal sepsis in full-term neonates. We found that CRP/MPV ratio was significantly higher in the sepsis group compared to the control group. CRP/MPV ratio showed a very high ability in predicting septic neonates. We believe that the CRP/MPV ratio may offer an advantage in the early diagnosis of neonatal sepsis which could be superior to either CRP or MPV when evaluated alone. There is only one study in the literature evaluating the CRP/MPV ratio in children with pneumonia suggesting that the CRP/MPV ratio might be used in differentiating bacterial from viral pneumonia and prediction of complications [28]. Recently, CRP/albumin ratio is used as a potential marker for gram-negative bacteremia in late-onset neonatal sepsis [29].

## 5. Conclusion

The current study shows for the first time that both salivary IL-10 and CRP/MPV ratio showed statistically significant differences between neonates with late-onset sepsis and controls. CRP/MPV is an easily calculated ratio which could be used as a simple and accurate marker for the diagnosis of late-onset neonatal sepsis. Salivary IL-10 could serve as potential noninvasive biomarker for the diagnosis of late-onset sepsis in full-term neonates.

## Figures and Tables

**Table 1:** Comparison of biomarkers for late-onset sepsis between septic and control neonates.

	Sepsis (*n* = 35) Mean ± SD	Control (*n* = 35) Mean ± SD	*P* value
Total leucocyte count (/mm^3^)	13.3 ± 6.3	12.8 ± 4.3	0.8
Platelet count (10^3^/mm^3^)	152.3 ± 201	245.1 ± 70.8	0.012^∗^
MPV (fL)	11.2 ± 0.9	8.6 ± 0.4	<0.001^∗^
CRP (mg/L)	78.2 ± 34	3.3 ± 1.7	<0.001^∗^
CRP/MPV ratio	7.08 ± 3.3	0.4 ± 0.2	<0.001^∗^
Serum IL-10 (pg/ml)	316 ± 198	23.7 ± 14	<0.001^∗^
Salivary IL-10 (pg/ml)	220 ± 150	18 ± 9	<0.001^∗^

MPV: mean platelet volume; CRP: C-reactive protein; IL-10: interleukin 10.

**Table 2:** Comparison of biomarkers for late-onset sepsis between blood culture positive and negative septic cases.

	Culture positive (*n* = 22)	Culture negative (*n* = 13)	*P* value
MPV (fL)	11.1 ± 0.9	11.4 ± 0.6	0.388
CRP (mg/L)	83.6 ± 34	60 ± 31	0.09
CRP/MPV	7.6 ± 3.3	5.3 ± 2.8	0.067
Serum IL-10 (pg/ml)	330.7 ± 198	267 ± 199	0.304
Salivary IL-10 (pg/ml)	278 ± 185	203 ± 137	0.304

MPV: mean platelet volume; CRP: C-reactive protein; IL-10: interleukin 10.

**Table 3:** ROC curves for salivary and serum IL-10, MPV, and CRP/MPV ratio to predict late-onset sepsis.

Parameter	AUC	SE	95% CI	Cutoff value	Sensitivity (%)	Specificity (%)	PPV (%)	NPV (%)
MPV (fL)	0.999	0.001	0.99-1	≥9.2	100	94.3	94.6	100
CRP/MPV ratio	1	0.000	1-1	≥0.90	100	97.1	97.2	100
Serum IL-10 (pg/ml)	0.97	0.024	0.92-1	≥33.6	97.1%	80	82.9	96.6
Salivary IL-10 (pg/ml)	0.994	0.005	0.98-1	≥31	97.1	94.3	94.4	97.1

MPV: mean platelet volume; CRP: C-reactive protein; IL-10: interleukin 10; PPV: positive predictive values; NPV: negative predictive value.

**Table 4:** Correlation between salivary and serum IL-10 and other laboratory markers.

Variables	Salivary IL-10		Serum IL-10	
	*R*	*P* value	*R*	*P* value
TLC	-0.08	0.649	0.374	0.027∗
MPV	0.201	0.246	-0.284	0.099
CRP	0.356	0.036∗	0.39	0.021∗
CRP/MPV ratio	0.390	0.006∗	0.424	0.011∗

TLC: total leucocytic count; MPV: mean platelet volume; CRP: C-reactive protein.

## Data Availability

The data that support the findings of this study are available on request from the corresponding author. The data are not publicly available due to privacy or ethical restrictions.
